# Giant Inflatable Colon and Community Knowledge, Intention, and Social Support for Colorectal Cancer Screening

**DOI:** 10.5888/pcd10.120192

**Published:** 2013-03-21

**Authors:** Diana Redwood, Ellen Provost, Elvin Asay, Janie Ferguson, Judith Muller

**Affiliations:** Author Affiliations: Ellen Provost, Elvin Asay, Janie Ferguson, Judith Muller, Alaska Native Tribal Health Consortium, Anchorage, Alaska.

## Abstract

**Introduction:**

Colorectal cancer (CRC) is the second-leading cause of deaths from cancer in the United States. Screening decreases CRC deaths through early cancer detection and through removal of precancerous lesions. We investigated whether a health exhibit consisting of a giant inflatable colon was an effective educational tool to increase community members’ knowledge, intention, and social support for CRC screening and prevention.

**Methods:**

Alaska adults (N = 880) attending community events statewide from March 2011 through March 2012 completed a short survey to assess knowledge about CRC, intention to get screened, and level of social support before and after walking through a giant interactive model of a human colon. The survey used a combination of open-ended questions and a Likert scale, where 1 was “very unlikely,” 2 was “somewhat unlikely,” 3 was “neutral,” 4 was “somewhat likely,” and 5 was “very likely.” The model depicted CRC stages from normal tissue to advanced adenocarcinoma and displayed signs with CRC prevention tips. We used the McNemar test and paired sample *t* tests for univariate analyses.

**Results:**

Respondents significantly improved their CRC knowledge (*P* < .05), intention to get screened (mean score increased from 4.3 to 4.5, *P* < .001), and comfort with talking to others about CRC screening (mean level of comfort increased from 3.8 to 3.9, *P* < .001). Multivariate analysis showed no significant differences by sex, age, or race for improvements in CRC screening knowledge, intention, or comfort.

**Conclusion:**

Interactive exhibits can improve public knowledge and interest in CRC screening, which may lead to increased CRC screening rates and decreased CRC incidence and deaths.

## Introduction

Colorectal cancer (CRC) is the second leading cause of cancer deaths in the United States. In 2009, an estimated 150,000 people were diagnosed with CRC, and 50,000 died from the disease (1). Among Alaska Native people, CRC is the leading cause of new cancer diagnoses and the second leading cause of cancer deaths. The age-adjusted CRC incidence and death rates for Alaska Native people are nearly twice those of US whites (2). An analysis of diagnosis by stage from 2005 to 2009 found that 57% of CRCs in Alaska Native people and 53% in US whites were diagnosed at a late stage (3).

Screening tests, including colonoscopy, flexible sigmoidoscopy, and fecal occult blood tests, can detect CRC in its early stages when it can be treated more effectively (4,5). Some tests, such as colonoscopy, can help prevent CRC because precancerous growths can be removed during the procedure. However, screening for this disease is underused, especially among people with low income, people who are underinsured or uninsured, and minorities (5,6). Alaska Behavioral Risk Factor Surveillance System (BRFSS) weighted population data from 2008 showed that 58% of Alaskan adults aged 50 years or older had ever had a colonoscopy or sigmoidoscopy (6). Rates varied by racial/ethnic group; 60% of Alaska whites reported ever having had either of these tests, compared with 51% of the Alaska Native population. Among Alaskans, 16% of adults in the same age group reported having used a blood stool test within the past 2 years (17% of whites and 10% of Alaska Native people).

Systematic, evidence-based reviews by the US Community Preventive Services Task Force outlined in the *Guide to Community Preventive Services* (www.thecommunityguide.org) have identified effective public health strategies that support increased CRC screening. The *Community Guide* recommends using small media (eg, brochures, videos), patient and provider reminder systems, provider assessment and feedback, and reduction of structural barriers (eg, modifying hours of service to meet client needs, offering services in nonclinical community settings, assisting with transportation or dependent-care needs) as effective strategies for increasing CRC screening, although the evidence comes primarily from studies that used fecal occult blood tests as the screening method (7). Data from the National Health Interview Survey and the National Cancer Institute’s Health Information National Trends Survey (HINTS) show associations between CRC knowledge, awareness, and intention to screen and receiving screening (8–10).

Various state and tribal health organizations throughout Alaska have worked to increase CRC screening. Projects to reduce structural barriers in the Alaska Tribal Health System have included training rural midlevel providers (registered nurses, physician assistants, and nurse practitioners) in flexible sigmoidoscopy, providing itinerant colonoscopy and flexible sigmoidoscopy services at rural tribal health facilities, increasing patient navigator services, and conducting research studies to assess the efficacy of fecal stool tests in this population to increase available screening methods (11–13). State and tribal health organizations have also created multiple small-media community health education tools, including brochures, posters, videos, and health fair displays (14). These tools are increasingly designed to be interactive and engaging and include multimedia components, such as theater events, personal stories, and videos (15–17). Multimedia tools include Readers’ Theater scripts, which are 30- to 45-minute scripts exploring cancer screening and diagnosis for community members to read aloud and discuss (15), and digital stories, which are 2- to 3-minute videos that community members make to share their cancer stories (16,17).

One educational medium that the *Community Guide* does not address is interactive exhibits. Such exhibits have been increasingly used by general and science museums to promote public participation and engagement (18). However, limited data exist on the extent to which interactive exhibits lead to greater understanding among viewers. Studies have primarily examined how people interact with the exhibits (19), not whether that interaction leads to increased knowledge and behavior change. A few studies have examined special events such as health fairs, community celebrations, educational parties, and Readers’ Theatre (15,20–24) as tools for behavior change. Examples of special events promoting increased access to health services and cancer screening include breast health educational parties, where games about breast health knowledge led to increased screening among underserved and uninsured women in New Jersey (20); a project that provided limousine service to and from screenings to encourage mammography use (21); cultural events such as the ‘Ohana Day Project in Hawaii (22); and health fair events to promote awareness and increase knowledge (23,24). In Alaska, a CRC-focused script was developed for use in Alaska Native communities as Readers’ Theatre and shown to increase comfort with cancer screening and interest in making health changes among theater participants (15). No studies have been published to date on the use of giant inflatable interactive colon exhibits as a strategy for promoting CRC screening and prevention. The objective of our study was to assess the use of such an exhibit for increasing knowledge of CRC screening and prevention in Alaska communities.

## Methods

In 2007, the Colossal Colon, a 40-foot-long molded crawl-through colon created by the Prevent Cancer Foundation (formerly the Cancer Research and Prevention Foundation), was exhibited in Alaska. Two local hospitals sponsored the display’s cost of $20,000 for 1 week in 2 Anchorage, Alaska, locations. Although the display was popular among the public, setting up the display was expensive and it was not wheelchair accessible. In 2009, the Prevent Cancer Super Colon, also created by the Prevent Cancer Foundation, was exhibited in Alaska for 4 days at the annual Alaska Federation of Natives meeting. The display cost of $12,000 was sponsored by Sanofi-Aventis. The Alaska Comprehensive Cancer Control Program attempted to purchase a Super Colon, but it was only available for rental. Program staff then researched potential colon displays for purchase and found a company (Landmark Creations International, Inc, Burnsville, Minnesota [www.landmarkcreations.com] that produced inflatable displays, including giant colons.

In October 2010, the Alaska Native Tribal Health Consortium Comprehensive Cancer Control Program in collaboration with the statewide CRC Partnership purchased an inflatable, flame-retardant, vinyl-coated nylon colon model from Landmark Creations (Figure). The model, nicknamed “Nolan the Colon,” cost $6,000 (including blower, tethers, installation hardware, repair kit, and storage bag). The interactive model is a walk-through replica of a human colon that measures 20 feet wide by 32 feet long by 14 feet high. Seven display signs on the walls of the model describe the development of cancer as a progression from normal tissue to metastatic cancer. The model uses raised relief and dark-red shading to depict polyps as abnormal colon tissue. The display sign for polyps explains that polyps are growths in the lining of the colon that usually are not cancerous but can become cancerous over time. The display depicts metastatic cancer as a giant mass that extends from inside the colon and wraps around to the outside of the colon wall; its sign explains how cancer cells travel and spread throughout the body via the lymphatic system. The display signs also provide tips and advice for CRC prevention, including quitting tobacco use, eating fruits and vegetables, being physically active, and obtaining regular colorectal screenings. Medical professionals in the Alaska Tribal Health System reviewed the initial model design and signage; Landmark Creations provided and modified them for clinical and cultural accuracy. Display signs advised viewers aged 50 or older (or younger if they had a family history of the disease) to obtain colon cancer screenings but did not recommend any specific screening method.

**Figure:**
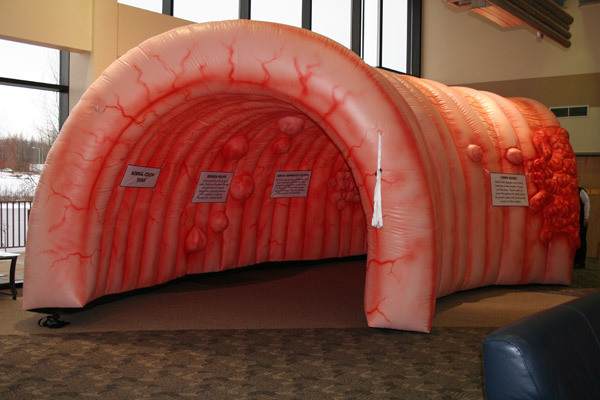
“Nolan the Colon” giant interactive colon model. The model is a walk-through inflatable replica of a human colon that illustrates the development of colorectal cancer. Viewers of this interactive exhibit gain a better understanding of how colorectal cancer is identified, how it advances, and how to lower their risk for the disease.

The purpose of the colon model is to increase CRC screening among Alaska community members statewide by increasing knowledge, intention to be screened, and comfort regarding CRC screening. Staff encouraged community members to physically interact with the giant colon to increase comfort and familiarity with the colon and understanding of cancer progression.

From March 1, 2011, through March 22, 2012, we exhibited the model in 14 rural and urban Alaska communities in 7 of the 12 major regions of Alaska and in 1 community in Canada. We displayed the model at 23 community events, including health fairs, basketball tournaments, arts-and-crafts fairs, and tribal gatherings. We surveyed Alaskan community members attending CRC prevention exhibits that included the colon model. The Alaska Area Institutional Review Board and the Alaska Native Tribal Health Consortium and Southcentral Foundation research and ethics committee reviewed and approved the study protocol.

### Survey instrument

The survey consisted of 4 knowledge questions, 2 intention questions, and 1 social support question. We adapted our questions from the 2003 and 2005 HINTS except for the family history question and the social support question (25). On the basis of our previous work with Alaska Native community members, we identified these 2 questions as important for this population. The first knowledge question asked at what age a person should have their first screening for CRC (age 30, 40, 50, or 65), the second asked whether removing a polyp from the colon could prevent cancer (yes/no; HINTS question #MM-05d), the third asked whether CRC always has symptoms that can be felt (yes/no; HINTS question # MM-05l), and the fourth asked whether a person with a family member with CRC is at higher risk of having it (yes/no). The 2 intention questions asked whether participants had ever been screened previously (yes/no) and how likely they were to get screened for CRC (5-point Likert scale where 1 was “very unlikely,” 2 was “somewhat unlikely,” 3 was “neutral,” 4 was “somewhat likely,” and 5 was “very likely” to be screened. [HINTS question #CC-22 and CC-26a]). Participants were asked on the presurvey if they had been screened but not asked again on the postsurvey. The social support question asked how comfortable the participant was talking to people about CRC screening (5-point Likert scale ranging from very unlikely to very likely). We also collected 3 demographic variables: age group (younger than 35, 35–49, 50–64, and ≥65), race/ethnicity (Alaska Native/American Indian/Aboriginal Canadian, white, other), and sex.

### Data collection

At each Alaska community event where we displayed the interactive health exhibit, we invited adult community members aged 18 years or older who walked through the colon model to complete a short, anonymous paper-and-pencil survey. An estimated 1,870 community members (range, 25–400 per event) viewed the colon model at 23 community events; approximately one-quarter of these viewers were younger than 18. Adult participants filled out the front side of the questionnaire before entering the display, viewed the exhibit, and filled out the back side of the questionnaire after exiting the exhibit. We considered surveys complete if participants answered at least 1 of the pre and post questions (eg, presurvey comfort with screening and postsurvey comfort with screening). We entered all survey respondent names into a drawing for a CRC screening promotional T-shirt or a berry-picking bucket.

### Statistical analysis

We compared the demographic characteristics of study participants with US Census 2010 data for all Alaskans (26), and we used the McNemar test and paired sample *t* tests for univariate analyses. We used multiple linear regression to determine demographic factors (age, sex, and race/ethnicity) associated with improvements in CRC screening knowledge, intention to get screened, and comfort talking with others about screening. The interaction between the 3 demographic variables — age, sex, and race/ethnicity — were not significant when included stepwise or in total in the regression models; therefore, we included only main effects in the final models. For the regression models we collapsed race/ethnicity into 2 groups (Alaska Native/American Indian/Aboriginal Canadian and white/other), and age group into under 50 and 50 or older. All analyses were 2-tailed, and we considered *P* < .05 to be significant. Data from respondents who did not answer both the pre and the post question were excluded from the analysis for that question. Of the 880 people who answered the survey, 759 (86%) answered at least 1 set of the pre/post questions, and 457 (52%) answered all questions (total response rate varied by question). All analyses were conducted with SPSS for Windows, Version 16.0 (SPSS Inc, Chicago, Illinois).

## Results

Of the approximately 1,400 adult community members who viewed the colon model, 880 (63%) responded to the survey. Almost three-quarters (71%) of respondents were female (Table 1); 31% were under age 35, and 12% were aged 65 or older. More than half (56%) of respondents were white; 37% reported their race as Alaska Native/American Indian/Aboriginal Canadian alone or in combination, and 7% reported their race as other. The overall screening rate was 35%, and of those who were age appropriate (age 50 or older), 64% had been screened for CRC previously. Compared with US Census 2010 data for Alaska residents, survey respondents were more likely to be female, over age 50, and Alaska Native/American Indian/Aboriginal Canadian.

**Table 1:** Demographic Characteristics and Responses, Nolan the Giant Colon Survey Respondents, 2011–2012, Compared With Alaska Residents, US Census, 2010

Characteristic	No. of Respondents (% [95% CI])	Alaska Census 2010^a^, No. of Respondents (%)
Total	880 (100)	710,231 (100.0)
**Sex**		
Male	229 (29 [25.7–32.0])	369,628 (52.0)
Female	564 (71 [68.0–74.3])	340,603 (48.0)
**Age, y**		
<35	246 (31 [27.8–34.2])	365,384 (51.4)
35–49	209 (26 [23.3–29.4])	147,700 (20.8)
50–64	242 (31 [27.3–33.7])	142,209 (20.0)
≥65	97 (12 [9.9–14.5])	54,938 (7.7)
**Race/ethnicity**		
Alaska Native/American Indian/Aboriginal Canadian alone or in combination	294 (37 [33.9–40.7])	138,312 (19.5)
White	439 (56 [52.2–59.2])	473,576 (66.7)
Other	55 (7 [5.2–8.8])	98,343 (13.8)

Abbreviation: CI, confidence interval.

a US Census Bureau, 2010 Census Summary File 1 (26).

Community member interaction with the colon model led to significant increases in all 3 domains: knowledge, screening intention, and social support. Survey responses demonstrated significant gains on all 4 CRC knowledge questions (*P* < .05) (Table 2). Responses also showed a significant increase in mean score for intention to be screened: from 4.3 to 4.5 (*t*_770_ = −5.73, *P* < .001). In the social support domain, respondents improved their comfort with talking to others about CRC screening (presurvey mean comfort, 3.8; postsurvey mean comfort, 3.9; *t*_758_ = −4.49, *P* < .001). There were no significant differences by sex, age, or race/ethnicity for the 3 domains.

**Table 2:** Responses of Participants, Nolan the Giant Colon Survey, 2011–2012

Question Category	Pre-visit, %	Post-visit, %
**Knowledge^a^**		
At what age should a person have their first screening for colorectal cancer? (Answered correctly, age 50)	65.1	94.0
Removing a polyp from your colon can prevent cancer. (Answered correctly, yes)	80.9	94.5
Colorectal cancer always has symptoms that you can feel. (Answered correctly, no)	92.4	94.7
If you have a family member with colon cancer, you are at a higher risk of having it too. (Answered correctly, yes)	87.6	92.4
**Intention**		
Ever screened previously? (n = 865)		
Yes (n = 304)	35.1	NA
No (n = 561)	64.9	NA
Likelihood of getting screened, mean score (n = 771)^b^	4.3	4.5
**Social Support**		
Comfort talking about CRC (1-5 scale), avg (n = 759) b	3.8	3.9

Abbreviation: NA, not applicable.

a Difference significant at *P* < .05; calculated using McNemar test.

b Mean difference significant at *P* < .05, calculated by using paired samples *t* tests for mean change. Scores based on Likert scale (range, 1–5); higher responses indicate higher likelihood.

## Discussion

The “Nolan the Colon” exhibit improved public knowledge and interest in CRC screening. Previous studies have noted barriers to increasing cancer screening among underserved minorities, including among Alaska Native people (14,27,28). Both Alaska Native and non-Native community members increased their knowledge, intentions, and social support for CRC screening and prevention through use of the interactive colon model. Giant inflatable colons are being used increasingly to promote CRC screening and awareness. The company that created the giant inflatable model used in this study has made 28 other colon models that are being displayed in 14 states. According to Landmark Creations (personal communication, November 2012), purchasers of colon models include medical centers, university teaching hospitals, state and tribal cancer prevention programs, and cancer prevention advocacy groups like Colon Cancer Alliance and the American Cancer Society. To our knowledge this is the first evaluation of the use of a giant inflatable colon for CRC screening education in a community setting.

The colon model that we used was a cost-effective teaching tool, with a per-event cost of $260 for the 23 community events in this study. Since that time, the colon model has been viewed at an additional 16 events, for a total per-event cost of $155, not including shipping and handling costs for transport across Alaska. The model will last for 5 to 10 years, so the expected per-event costs will continue to decline as it is used in the future.

This study has several limitations. The response rate for the survey was generated from staff estimates of the number of event attendees; we did not conduct a formal count of attendees at each location. However, all adult community members who viewed the colon model were invited to participate. A higher proportion of women than men viewed the colon model, so a higher proportion of women completed the survey (71% women vs 29% men). However, when possible, we reported sex-specific data. Because the focus for this cancer education intervention was to increase screening among rural populations, we did not conduct enough surveys in urban areas (3%) to stratify results based on region. Additionally, although community members who viewed the colon model significantly increased their knowledge, intention to screen, and comfort with screening, we are unable to determine from these data the percentage of community members who obtained CRC screening after viewing the colon exhibit. This is especially important for the 36% of respondents who were age-appropriate for screening but said they had not been screened previously. Further research is necessary to assess screening completion behaviors post-exhibit. Furthermore, our results may be subject to social desirability response bias because of the short time period between the presurvey administration and the postsurvey, which may have led some viewers to complete the postsurvey more carefully than they would have otherwise to show that they had improved from the presurvey. Finally, we were unable to find any other evaluations of the use of these models for health promotion, only information on how to rent or purchase these models or how to use them in communities.

Alaska BRFSS Prevalence and Trends Data (http://apps.nccd.cdc.gov/brfss/) show an increase over the past 10 years in the proportion of the population aged 50 or older who report ever having a sigmoidoscopy or colonoscopy. In 1997, only 41% of Alaskans reported ever having a colonoscopy or flexible sigmoidoscopy. In 2010, before the start of our study, the Alaska BRFSS screening rate was 62%; the rate increased to 65% in 2011. Over the previous 15 years, CRC screening rates have been increasing statewide, as have multiple prevention activities, including the giant colon exhibits. However, the screening rate is less than the Centers for Disease Control and Prevention (CDC) goal of 80% of adults aged 50 or older by 2014, which suggests that further efforts to increase screening are warranted.

Our evaluation data suggest that the giant inflatable colon is a promising community-level intervention that provides a novel population-based strategy for increasing CRC screening and prevention. Furthermore, the colon model appeared to be equally effective for men and women, people of all ages, and Alaska Native and non-Native community members in all 3 domains surveyed (knowledge, intention, and social support). This tool encourages CRC screening and could ultimately help to decrease illness and death from this preventable disease.
